# Supplementary material for: Potentially Suitable Geographical Area for Monochamus alternatus under Current and Future Climatic Scenarios Based on Optimized MaxEnt Model
Source: Insects. 2023 Feb 13;14(2):182. doi: 10.3390/insects14020182 (PMC9962367; doi:10.3390/insects14020182)
Supplement: Supplementary file 1 [file insects-14-00182-s001.zip › insects-2183928-supplementary.pdf]

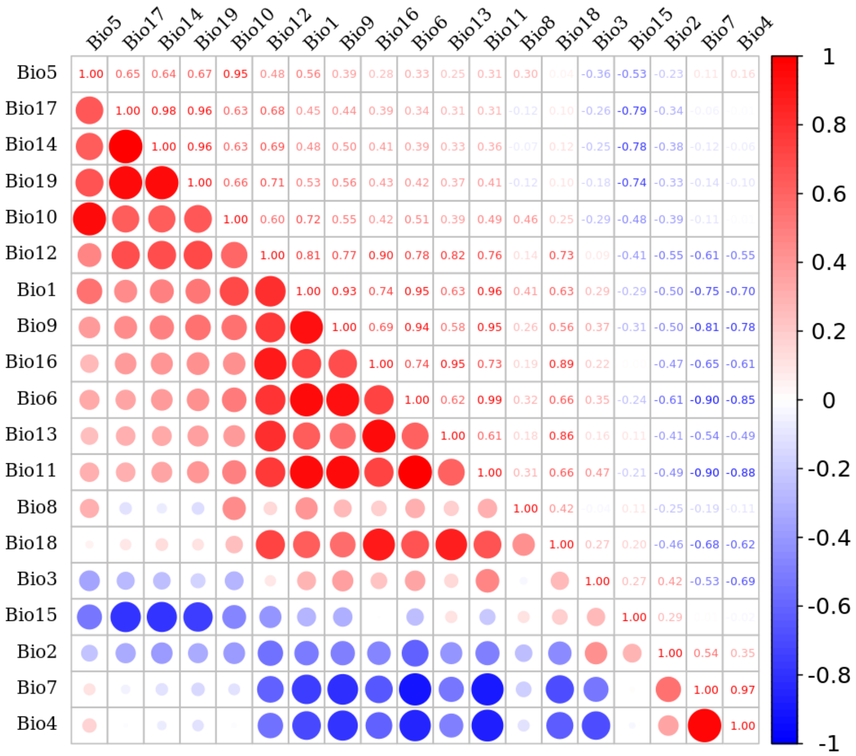

Figure S1. Pearson correlation analysis and correlation coefficients of 19 bioclimatic variables. The color of red means positive correlation, blue means negative correlation. The size of the circle means the strength of the correlation. Bio1 represents the Annual mean temperature, Bio2 represents the Monthly mean temperature difference, Bio3 represents the Isothermality, Bio4 represents the Temperature seasonality, Bio5 represents the Max temperature of warmest month, Bio6 represents the Min. temperature of the coldest month, Bio7 represents the Temperature annual range, Bio8 represents the Mean temperature of the wettest quarter, Bio9 represents the Mean temperature of driest quarter, Bio10 represents the Mean temperature of warmest quarter, Bio11 represents the Mean temperature of coldest quarter, Bio12 represents the Annual precipitation, Bio13 represents the Precipitation of wettest month, Bio14 represents the Precipitation of driest month, Bio15 represents the Precipitation seasonality, Bio16 represents the Precipitation of wettest quarter, Bio17 represents the Precipitation of driest quarter, Bio18 represents the Precipitation of warmest quarter , Bio19 represents the Precipitation of coldest quarter.

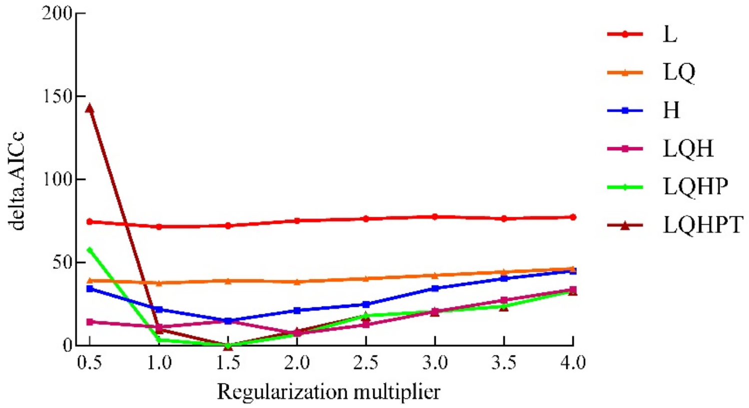

Figure S2. The ΔAICc values of the 48 different combinations of the MaxEnt parameter settings.

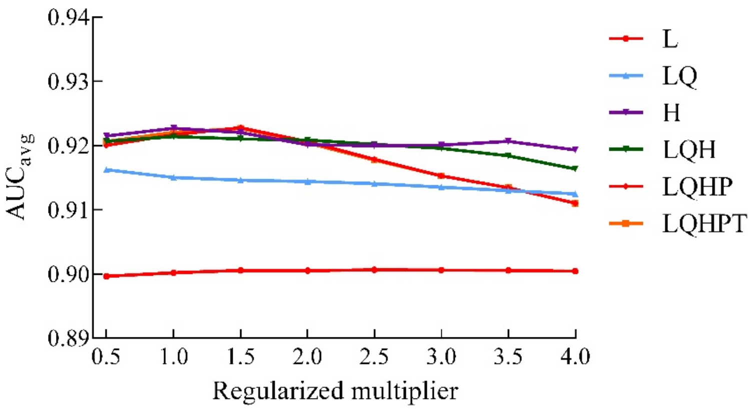

Figure S3. The AUC_{avg} values of the 48 different combinations of the MaxEnt parameter settings.

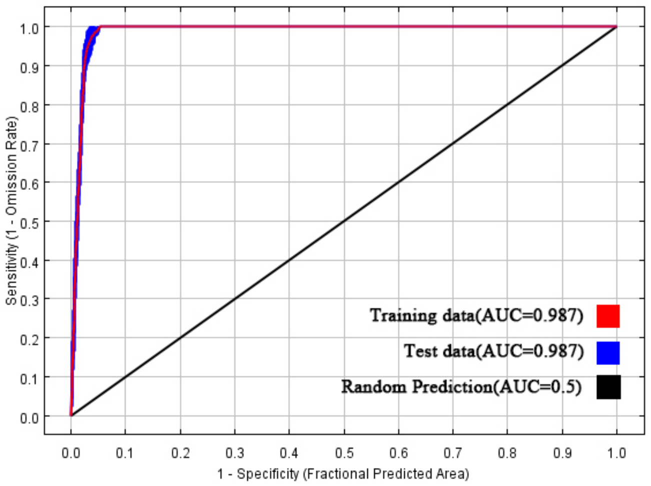

Figure S4. ROC curve and the values of AUC for the optimized MaxEnt model.

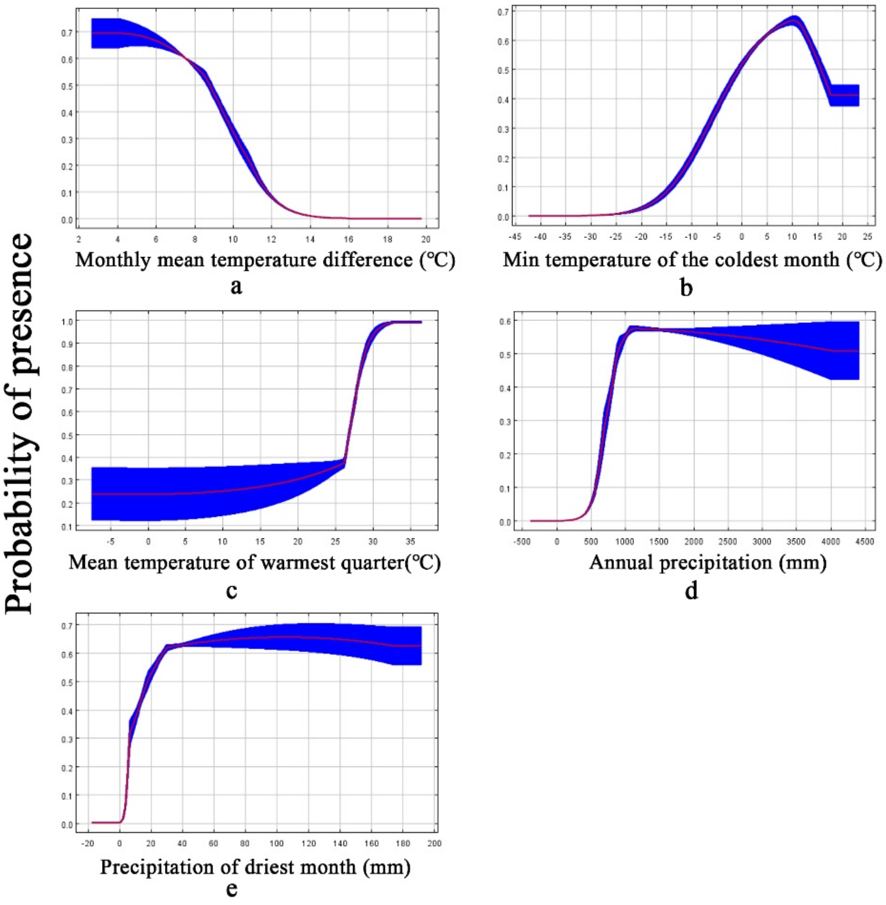

Figure S5. Response curves of the bioclimatic variables most related to the distribution of *M. alternatus*. (a) Response curve of monthly mean temperature difference (Bio2), (b) Response curve of minimum temperature of the coldest month (Bio6), (c) Response curve of mean temperature of warmest quarte (Bio10), (d) Response curve of annual precipitation (Bio12), (e) Response curve of precipitation of driest month (Bio14).

Table S1. The contribution rates of bioclimatic variables affecting the distribution of *M. alternatus*.

Code	Bioclimatic Variables	Contribution rate/%
Bio14	Precipitation of driest month	46.4
Bio12	Annual precipitation	11.2
Bio6	Min. temperature of the coldest month	9.2
Bio10	Mean temperature of warmest quarter	7.2
Bio5	Max temperature of warmest month	5.2
Bio4	Temperature seasonality	4.8
Bio8	Mean temperature of the wettest quarter	2.8
Bio1	Annual mean temperature	2.7
Bio2	Monthly mean temperature difference	1.6
Bio19	Precipitation of coldest quarter	1.4
Bio15	Precipitation seasonality	1.3
Bio16	Precipitation of wettest quarter	1.1
Bio11	Mean temperature of coldest quarter	1
Bio7	Temperature annual range	1
Bio17	Precipitation of driest quarter	0.8
Bio9	Mean temperature of driest quarter	0.8
Bio3	Isothermality	0.8
Bio18	Precipitation of warmest quarter	0.4
Bio13	Precipitation of wettest month	0.3
